# Factors associated with homeless experiences amid the COVID-19 pandemic in the Nipissing District, Ontario, Canada

**DOI:** 10.1371/journal.pone.0305485

**Published:** 2024-07-24

**Authors:** Megan Odd, Amir Erfani

**Affiliations:** 1 Centre of Access, Interdisciplinary Studies and Lifelong Learning, Canadore College, North Bay, Ontario, Canada; 2 Department of Sociology, Nipissing University, North Bay, Ontario, Canada; Dayeh University, TAIWAN

## Abstract

Canadian homelessness is an ongoing issue, especially in the Nipissing District, Ontario, where agencies work to support those in need. However, these efforts were challenged with the sudden onset of the COVID-19 pandemic. Drawing on the *Cycle of Homelessness* model, this study examines sociodemographic factors associated with homeless experiences during the pandemic. Using data from the 2021 (n = 207) Nipissing District homeless enumeration survey and employing bivariate and multivariate binary logistic analyses, this study examined sociodemographic factors associated with reasons of homelessness, barriers to housing loss and experiences of chronic and episodic homelessness during the pandemic. The results showed a significant sociodemographic variation in the experiences of the homeless population during the COVID-19 pandemic. Those over the age of 35 versus their younger counterparts were more likely (43.7%) found in emergency shelters. Multivariate findings indicated that females experienced housing/financial loss and interpersonal/family issues, directly causing homelessness, 2.2 and 2.5 times more than males, respectively. Welfare recipients were more likely to experience health-related reasons for housing loss (Odds Ratio (OR): 2.8), chronic homelessness (OR: 3.3), addiction (OR: 2.9), and mental health-related barriers to housing (OR: 4.1). Those aged 25–34, 25–44, and 45+ were 7.9, 4.9, and 5.1 times more likely to face chronic homelessness.

**Conclusions**: Welfare recipients are more at-risk of health-related housing loss, addiction, and mental health barriers to housing, and chronic homelessness. This could be attributed to poor public planning and policies that put people in marginal economic and housing circumstances, especially during the pandemic. Therefore, policy reform is required to address the main barriers in eliminating homelessness.

## Background

Homelessness is an ongoing issue at the national, provincial, and municipal levels in Canada [1], especially with the recent COVID-19 pandemic. Defined as, “the situation in which an individual has no stable, permanent, and appropriate housing, or an immediate prospect of” [2], homelessness varies by geographic location based on sociodemographic and key causal factors [2]. Specifically in the District of Nipissing, Ontario, fluctuations have been observed in average age, gender composition, Indigenous makeup, shelter usage, reasons for housing loss and barriers to obtaining safe and affordable housing [2–7]. To combat these changes and address the increasing homeless population, local service agencies provide a range of services and support for those in need, including welfare programming and housing services [8].

These efforts, however, have been challenged with recent shortages in affordable, safe, and stable housing [9]. It is estimated that the average cost of Canadian housing has increased by 20% since the beginning of the COVID-19 pandemic (2020) [10]. This is not the only systemic shortfall. The lack of adequate services and supports, as well as system care has been documented to increase the risk of vulnerable populations to join, or return to, the homeless sector [10]. Accompanying the pandemic were reduced shelter capacities, staffing shortages, and sudden closures of services and supports [11]. These changes, considered as *social exclusion*, “the structural policies and processes that have systematically excluded people from economic, social, cultural, and political processes”, have been highlighted as precipitators to homelessness [11]. Apart of these structural factors of homelessness, there are little knowledge on individual-level factors associated with homelessness during the recent pandemic in Northeastern Ontario, Canada. Therefore, this study aims to examine sociodemographic risk factors associated with homeless experiences of homeless population in the Nipissing District of Ontario province in Canada during the COVID-19 pandemic.

## Theoretical background and previous findings

### The cycle of homelessness

During the 1980’s, researchers investigating the causes of homelessness were divided, emphasizing either ‘social structural’ or ‘individual’ level factors of homelessness [12, 13]. As research advanced, many recognized that both play a key role. In 1988, Wolch and colleagues combined these two perspectives and developed a conceptual framework explaining the pathway into homelessness. They foresaw homelessness as the “end state of a long, complex, social, and personal process” [14:443]. They stressed that homelessness is not a sudden event, but rather the “culmination of a long process of economic hardship, isolation, and dislocation” [14:443]. The authors’ called this, the *Cycle of Homelessness*. In the first stage, it is argued that there are pre-existing structural factors that increase the number of people who live in marginal and economic housing circumstances. They label this group, the “potentially homeless”. The second stage proposes that adverse life events propel these individuals into homelessness. The third and final stage of the model is life in homelessness itself. Wolch and colleagues [14] add that this state aggravates the social and environmental conditions that put individuals at-risk, and hence diminish their ability to escape. Chamberlain and Johnson [15] put forth the argument that social adaptation also takes place for those in homelessness, as they become accustomed to the “way of life”, further supporting Wolch’s ideas. This cycle can lead individuals to a state of chronicity, where they remain homeless for more than 180 days (or 6 months) of a give one-year period [16]. In the following, the literature examining the causal factors of homelessness, identified in the three stages of the discussed conceptual model will be reviewed.

### Structural factors

There exist, structural factors that create catalyzed environments for at-risk individuals including the lack of safe and affordable housing, system failure, and systemic discrimination. These factors precede homelessness in Canada [17–19]. Since the beginning of the COVID-19 pandemic, available housing options to those in need are expensive and often not accessible to those on financial welfare programs or working part-time [17]. The increasing cost of Canadian housing (20%) has also outpaced the growth of both employment and/or government supported incomes [19]. This lack of accessible housing has been identified as leading to a range of short and long-term effects, including homelessness [18].

Social exclusion, defined as the “structural policies and processes that have systematically excluded people from economic, social, cultural and political processes”, has also been identified as a main precipitator to homelessness [20]. At the district-level, similar structures have been observed. The Northeast region of Ontario, in which the Nipissing District is situated, has faced issues with social assistance where current welfare amounts are inadequate or individuals in need do not qualify [17].

Other research has revealed high unemployment rates and flawed social programming to be at the core of homelessness in regions surrounding the District of Nipissing [21]. Business closures during the pandemic predominantly affected certain sociodemographic groups, which we have examined in the following section.

### Sociodemographic risk factors

Through the *Cycle of Homelessness* lens, specific risk factors for homelessness can present themselves in the form of both past experiences and sociodemographic characteristics. Mental health and substance use have a two-way causal relationship with homelessness. Several studies have shown that those experiencing homelessness are susceptible to chronic mental illness and substance abuse issues, often accompanying each other [15, 21, 22]. Also, those struggling with severe and persistent mental health and/or substance abuse are more likely to experience chronic homelessness [23, 24].

Marital breakdown and abusive relationships also precede homelessness. Those experiencing the dissolution of a long-term relationship are at-risk of economic insecurity, as well as jeopardized housing as a result [24, 25]. Researchers have found that those who have separated from, or divorced their partners are twice as likely to experience homelessness [26]. These separations can result from a gradual dissolution of an abusive relationship, or an amicable split.

Childhood adversity has been the focal point of homelessness research dating back to 1995. These studies have found that adverse childhood experiences (ACEs) are frequently present among the adult homeless population [26–30]. In 1997, these ACEs were categorized into sub-groups: lack of care, physical abuse, and sexual abuse [31].

Socioeconomic status (SES) is inversely related to individual-level susceptibility to homelessness. Evidence shows that individuals with low SES are more likely to become homeless [13, 15, 24, 30, 32–35]. One study reported that poverty is among the factors that are disproportionately present in the background of homeless adults [30]. The developing trend that demographers are seeing, is that individuals are being forced to spend larger proportions of their annual income on housing. When this exceeds 30%, these individuals become susceptible to housing loss [35]. The “social” prefix to SES includes the social capital that a person holds. Studies found that those with weak social ties face higher rates of homelessness [22, 36], specifically that 36% of the population had no friends and 31% had no connection with family members. This social deprivation removes the possibility of both social and economic support.

Indigenous peoples are considered one of the most vulnerable sub-groups of the Canadian homeless population. It is noted that this group is twice as likely to experience a period of homelessness during their lifetime [26]. Various studies have shown high rates of Indigenous homelessness, despite accounting for a minimal (4.3%) proportion of the national population [31]. National and regional research has revealed mass overrepresentation of Indigenous peoples, with 30% and 26% of the total homeless population respectively [31, 37]. As one of the most “materially, socially, and deprived ethnocultural group in Canada” [38], the Indigenous populations experience homelessness due to “historical dispossession of lands, colonial and neocolonial practices of cultural oppression and erosion, intergenerational traumas, systemic racism, government policies”, and “the current economy and housing markets” [38].

### Adverse life events

Adverse life events are described as those that consequently launch an individual into a state of homelessness [14]. These risk factors can be divided into four sub-groups: financial crises, interpersonal conflict, transitions from institutionalized care, and abrupt social change.

Financial crises can result from loss of employment and/or the rapid increase of housing and living expenses [22, 23, 28, 35]. Past studies have shown a 75% unemployment rate among the homeless population [22]. The duration of homeless periods was also found to be longer than the time these individuals had been unemployed, confirming the causal relationship between job loss and homelessness [22]. Although there are “social policies that provide financial and other support to individuals living in poverty” [23], only a small portion of eligible applicants receive them. Recently, social assistance amounts provided to low-income Canadians has been decreasing [24], and these continual reductions are precursors to homelessness.

Interpersonal conflict has also been found to increase the likelihood of homelessness, through various means including marital breakdown, intimate partner violence, youth-parent conflict, and tenant-landlord conflict. As mentioned, the abrupt or sudden termination of a relationship to escape intimate partner violence can lead to housing loss. One study found homelessness as a common result of fleeing maltreatment, with 106,000 women and children admitted to violence against women (VAW) shelters in 2002 [24]. More recently, 20% of surveyed homeless women reported having been physically or sexually abused within six months prior to their loss of housing [28]. Like amicable break-ups, the separation from a partner may lead to economic insecurity and jeopardized living situations [24, 39]. Interpersonal conflicts are not isolated to romantic partners. Youth-parent conflict also frequently leads to homelessness. In 2012, between 30–50% of homeless youth cited family problems as the main reason for leaving home [29]. Conflicts between tenant and landlord occur when tenants are not able to abide by set rules, regulations, and expectations as provided by the landlord, or from an increase in rental and living expenses resulting in eviction [35].

Abrupt social changes, such as those resulting from the COVID-19 pandemic can also be considered catalysts for homelessness. Researchers questioned how this highly contagious virus would affect levels of homelessness at the national, provincial/territorial, and municipal levels. In 2020, homelessness was declared a major public health concern, even more so during a pandemic [40]. “Infection disease epidemics and pandemics have a disproportionate impact on people experiencing poverty marginalization, stigmatization, and discrimination” including those in homelessness [40]. Homeless shelters have also been identified as ideal environments for illness transmission, which can be accredited to shared living spaces, overcrowding, and the inability to distance from others [40]. The pandemic has not only increased vulnerability of the homeless population through contracting COVID-19, but in the closure of services and supports due to decreased staff availability [40]. The sudden closure or reduction of access to drop-in centres, community centres, and services can result in compromised physical and mental health [40]. The above evidence may assist researchers in determining the characteristics associated with homelessness amid the pandemic, however, little is known about this experience, specifically in the District of Nipissing. We address this gap by studying the sociodemographic composition of the homeless population and the correlates of their experiences. Drawing on the *Cycle of Homelessness*, we will examine sociodemographic profiles of the homeless population, reasons for housing loss, barriers to obtain and securing housing, and d the sociodemographic risk factors associated with reasons for housing loss, episodic homelessness, chronic homelessness, and barriers to housing during the COVID-19 pandemic in the District of Nipissing.

## Methods

### Data

This research used data from the most recent enumeration survey of homeless individuals conducted in October 2021, in communities located across the District of Nipissing, Ontario, Canada. The surveys were conducted by front-line workers and volunteers from various agencies in the Nipissing District who work directly with the homeless population. These agencies work collaboratively on the Nipissing District Housing and Homelessness Partnership, and with guidance of both the Canadian Alliance to End Homelessness and Built for Zero Canada. The surveys were completed on an opportunity-basis, where front-line workers asked their clients if they were willing to complete the survey.

The District of Nipissing Social Services Administration Board (DNSSAB), and affiliated community partners funded and conducted the 2021 enumeration survey using face-to-face interviews with homeless populations in real-time, using the Point-in-Time (PiT) count method. This method comprises a 24-hour period during which volunteers and service providers complete a physical count and survey of those experiencing homelessness in the District. On October 13, 2021, the survey teams visited homeless hot spots in the municipalities of North Bay, Mattawa, West Nipissing, Temagami, East Ferris, Chisholm, and South Algonquin. Through the interviews, a wide range of data were collected, including demographic characteristics, housing history, and barriers to housing. The 2021 survey yielded a 69.0% response rate (207/300). The DNSSAB granted access to the microdata survey file for this study’s use on February 8, 2022, and Nipissing University in Canada (File Number: 102855) provided ethic approvals for the Secondary Use of Data for this study.

### Independent and dependent variables

The independent variables are socioeconomic and demographic characteristics of homeless individuals, including age, age at first homelessness, gender, Indigenous status, experience in foster care, current source of income, and health status. Dependent variables are homeless experiences that include sleeping location, episodic homelessness (number of times each respondent experienced a homeless period over the last 12 months), chronic homelessness (cumulative amount of time spend homeless over the last 12 months), reason(s) for housing loss (financial, health, and interpersonal/family issues), and barriers to housing (addiction, family breakdown, mental illness, and stigma/discrimination). These variables are standard data points that the Ontario Government selected for the purposes of comparison across Districts in the province.

### Analysis

Bivariate analyses were conducted to determine which socioeconomic and demographic variables were significantly related to various experiences frequently documented by the homeless population. This includes sleeping location, reason for housing loss, frequency and duration of homeless periods, and barriers to housing. A chi-square test was employed, considering a p-value of.05 or lower to be statistically significant since the experiences were measured by categorical nominal or ordinal values.

Binary logistical multivariate regression analyses were used to examine the adjusted relationship between socioeconomic and demographic characteristics of homeless people and their experiences. Since the study sample was small, it was not possible to use multinominal logistic regression for the nominal dependent variables with more than two categories. Therefore, the dependent variables were converted into dummy variables that include 1) housing loss due to *housing/financial loss*, *health issues*, or *interpersonal/family issues* (Yes/No); 2) *chronic homelessness* (Chronic/Non-Chronic); and 3) barriers to housing due to *addiction*, *mental health*, *family breakdown*, and *stigma/discrimination* (Yes/No).

## Results

### Bivariate analysis

#### Sleeping location

The results in Table 1 showed that age and gender or homeless individuals was significantly related with their sleeping location. Those under the age of 35 were less likely to use emergency shelters (19.4%) compared to their older counterparts (43.7%). Young (<25) individuals slept mostly in transitional shelters (34.5%). Those under 35 were also more frequently found in surveilled institutions (49.3%). With respect to gender identity, male and female individuals used emergency shelters at equal rates. However, females were more likely to stay with someone they knew (20.6%) compared to males (11.5%) and were roughly five times more likely to stay in transitional shelters (17.7% vs. 3.1%). In contrast, males were more likely to be found in institutional settings (27.7% vs. 10.3%), and unsheltered locations (28.5% vs. 20.6%). Those who were in foster care during their youth were more likely to stay in emergency shelters than those who were not (18.2% vs. 16.4%), with friends or family (16.4% vs. 14.4%), institutional settings (23.6% vs. 20.5%) and in transitional shelters (16.4%).

**Table 1:** Percent distribution of homeless individuals by sleeping location, according to selected characteristics, Nipissing District, Ontario 2021.

Characteristics	Emergency shelter	Someone else’s place	Institutional setting	Hotel/motel funded by homeless program	Unsheltered	Transitional Shelter	Total (n)
**Age** ***							
<25	6.9	0.0	20.7	20.7	17.2	34.5	**29**
25–34	12.5	17.9	28.6	5.4	33.9	1.8	**56**
35–44	16.4	18.0	21.3	8.2	29.5	6.6	**61**
45+	27.3	18.2	12.7	18.2	18.2	5.5	**55**
**Gender** **							
Male	17.7	11.5	27.7	11.5	28.5	3.1	**130**
Female	17.6	20.6	10.3	13.2	20.6	17.6	**68**
**Age at first homelessness**							
Less than 25	13.9	9.9	27.7	10.9	27.7	9.9	**101**
25 and over	17.9	23.2	15.8	12.6	24.2	6.3	**95**
**Experience in foster care**							
Yes	18.2	16.4	23.6	7.3	18.2	16.4	**55**
No	16.4	14.4	20.5	13.7	29.5	5.5	**146**

Significant level of Chi-square test:

*** p ≤ .001,

** p ≤ .01,

* p ≤ .05

#### Reason for housing loss

*Housing/financial loss*. Gender (p ≤.05) and health status (p ≤.05) were statistically correlated with *housing/financial loss* as the main reason for homelessness (Table 2). Females were more likely thank males (42.6% vs. 26.9%) to report financial problems as their main cause of housing loss. In terms of overall health, those who reported fair health (46.8%) were most likely to experience housing and/or financial loss that resulted in homelessness, compared to those who reported good (27.5%) or poor overall health (21.2%).

**Table 2:** Percent distribution of homeless individuals by reason for housing loss, according to selected characteristics, Nipissing District, Ontario 2021.

Demographics	Housing/ Financial Loss^1^		Health Related^2^		Interpersonal/ Family Issues^3^		Total (n)
	No	Yes	No	Yes	No	Yes	
**Age**							
<25	10.9	3.5	82.8	17.2	31.0	69.0	**29**
25–34	16.9	10.9	69.6	30.4	58.9	41.1	**56**
35–44	18.9	11.4	91.8	8.2	55.7	44.3	**61**
45+	20.3	6.9	83.6	16.4	43.6	56.4	**55**
**P-value**	**.254**		**.019** *		**.054**		
**Gender**							
Male	73.1	26.9	53.0	12.6	55.4	44.6	**130**
Female	57.4	42.6	28.7	5.5	39.7	60.3	**68**
**P-value**	**.018** *		**.597**		**.036** *		
**Indigenous status**							
Non-Indigenous	39.1	15.9	45.4	9.6	25.1	29.9	**114**
Indigenous	28.0	16.4	36.7	7.7	23.6	20.7	**92**
**P-value**	**.373**		**.099**		**.341**		
**Health status**							
Poor	78.8	21.2	12.7	3.3	8.3	7.8	**33**
Fair	53.2	46.8	23.0	7.3	17.6	12.7	**62**
Good	72.5	27.5	46.0	7.3	22.5	30.8	**109**
**P-value**	**.012** **		**.207**		**.127**		
**Age at first homelessness**							
Less than 25	36.7	14.8	76.2	23.8	22.4	29.1	**101**
25 and over	30.6	17.8	87.4	12.6	25.0	23.4	**95**
**P-value**	**.225**		.044*		**.261**		
**Income source**							
Welfare	42.0	21.2	77.1	22.9	55.7	44.3	**131**
Non-welfare	25.6	11.1	90.8	9.2	36.8	63.2	**76**
**P-value**	**.622**		**.013** **		**.009** **		

^1^ includes Building Sold or Renovated, Complaint, Left Community, Not Enough Income for Housing, Owner Moved In, and Unfit/Unsafe Housing.

^2^ includes Hospitalization or Treatment Program, Mental Health, Physical Health, and Substance Abuse

^3^ includes Experienced discrimination, Conflict with tenant/landlord, Conflict with parent/guardian, Conflict with spouse/partner, Conflict with other, Death of family member, Departure of family member, Experienced abuse from parent/guardian, Experienced abuse from spouse/partner, Experienced abuse from other, and Family breakdown

Significant level of Chi-square test:

*** p ≤ .001,

** p ≤ .01,

* p ≤ .05

*Health-related*. Reported health reasons for housing loss were significantly related to *age* (p ≤.05), *age at first homelessness* (p ≤.05), *and income source* (p ≤.05) (Table 2). Specifically, those between the ages of 25 and 34 are most likely to experience health related housing loss (30.4%) along with individuals who became homelessness for the first time before the age of 25 (23.8%), and those who receive welfare income supports (22.9%). There were no significant differences between Non-Indigenous and Indigenous individuals in terms of their health-related reasons for housing loss.

*Interpersonal/family issues*. The results in Table 2 show that *gender* (p ≤.05), and *income source* (p ≤.01) were statistically associated with *Reason for Housing Loss*: *Interpersonal and/or Family Issues*. Females reported this reason for homelessness more frequently than males (60.3% vs. 44.6%). Also, those with non-welfare sources of income were more likely to experience this main cause for homelessness (63.2%), compared to individuals receiving welfare supports (44.3%).

#### Episodic homelessness

*Age of respondent* (p ≤.01) was the only variable significantly related to *episodic homelessness* (Table 3). The results showed that those aged 45 and above were more likely to experience only one episode of homelessness (84.0%) compared to their younger counterparts. Respondents under the age of 25 were predominantly found to experience two (20.8%) or more episodes (33.3%) of homelessness compared to other age groups.

**Table 3:** Percent distribution of homeless individuals by episodes of homelessness, according to selected characteristics, Nipissing District, Ontario 2021.

Demographics	Episodes of Homelessness			
	1 episode	2 episodes	3+ episodes	Total (n)
**Age** **				
<25	45.8	20.8	33.3	**29**
25–34	72.7	9.1	18.2	**56**
35–44	66.7	11.7	21.7	**61**
45+	84.0	10.0	6.0	**55**

Significant level of Chi-square test:

*** p ≤ .001,

** p ≤ .01,

* p ≤ .05

#### Chronic homelessness

Respondents’ *age* (p ≤.01) and *income source* (p ≤.001) were statistically related to *chronic homelessness* (Table 4). Those aged 25 to 34 were most likely (68.5%) to experience chronic homelessness, compared to other age groups. Those who received welfare income supports were twice as likely than those with other forms of income to experience chronic homelessness (63.6% vs. 35.4%).

**Table 4:** Percent distribution of homeless individuals by duration of homelessness, according to selected characteristics, Nipissing District, Ontario 2021.

Demographics	Chronic Homelessness		
	Non-chronic	Chronic	Total (n)
**Age** **			
<25	78.3	21.7	**29**
25–34	31.5	68.5	**56**
35–44	49.1	50.9	**61**
45+	47.9	52.1	**55**
**Gender**			
Male	42.4	57.6	**130**
Female	56.7	43.3	**68**
**Income source** ***			
Welfare	36.4	63.6	**131**
Non-welfare	64.6	35.4	**76**

Significant level of Chi-square test:

*** p ≤ .001,

** p ≤ .01,

* p ≤ .05

#### Barriers to housing

*Addiction*. The results in Table 5 indicate that *age of respondent* (p ≤.05), *age at first homelessness* (p ≤.05), and *income source* (p ≤.01) were all significantly associated with *Barrier to Housing*: *Addiction*. In fact, those between the ages of 25 and 34 (37.5%) were most likely to have trouble finding housing due to their struggles with addiction, compared to other age groups. Males were also 11.5% more likely than females (27.7% vs. 16.2%) to experience this same difficulty. Those who experienced homelessness before the age of 25 rather than at 25 or over (31.7% vs. 17.9%), and those receiving welfare income supports (31.3%) were also more likely to face these struggles.

**Table 5:** Percent distribution of homeless individuals by barrier to housing, according to selected characteristics, Nipissing District, Ontario 2021.

Demographics	Addiction		Family Breakdown		Mental Illness		Stigma/ Discrimination		Total (n)
	No	Yes	No	Yes	No	Yes	No	Yes	
**Age**									
<25	75.9	24.1	75.9	24.1	69.0	31.0	75.9	24.1	**29**
25–34	62.5	37.5	91.1	8.9	83.9	16.1	76.8	23.2	**56**
35–44	75.4	24.6	91.8	8.2	91.8	8.2	67.2	32.8	**61**
45+	87.3	12.7	94.5	5.5	89.1	10.9	92.7	7.3	**55**
**P-value**	**.028** *		**.045** *		**.028** *		**.010** **		
**Gender**									
Male	47.4	18.2	94.6	5.4	55.0	10.6	53.0	12.6	**130**
Female	28.7	5.5	83.8	16.2	31.3	3.0	25.7	8.5	**68**
**P-value**	**.071**		**.012** **		**.154**		**.346**		
**Indigenous status**									
Non-Indigenous	41.5	13.5	90.4	9.6	85.1	14.9	44.4	10.6	**114**
Indigenous	33.8	10.6	90.2	9.8	87.0	13.0	33.3	11.1	**92**
**P-value**	**.214**		**.012** **		**.048** *		**.535**		
**Age at first homelessness**									
Less than 25	68.3	31.7	45.4	6.1	79.2	20.8	71.3	28.7	**101**
25 and over	82.1	17.9	44.3	4.0	91.6	8.4	84.2	15.8	**95**
**P-value**	**.026** *		**.424**		**.015** *		**.030** *		
**Health status**									
Poor	78.8	21.2	14.2	1.9	72.7	27.3	13.2	2.9	**33**
Fair	66.1	33.9	27.4	2.9	90.3	9.7	23.5	6.8	**62**
Good	80.7	19.3	48.5	4.9	88.1	11.9	41.6	11.7	**109**
**P-value**	**.091**		**.882**		**.043** *		**.872**		
**Income source**									
Welfare	68.7	31.3	57.4	5.7	52.6	10.6	48.7	14.4	**131**
Non-welfare	86.8	13.2	32.3	4.3	32.8	3.8	29.4	7.2	**76**
**P-value**	**.004** **		**.583**		**.217**		**.595**		

Significant level of Chi-square test:

*** p ≤ .001,

** p ≤ .01,

* p ≤ .05

*Family breakdown*. Age of respondent, gender identity, and Indigenous identity were significantly related to *Barrier to Housing*: *Family Breakdown* at the 95% confidence level (Table 5). Those under the age of 25 (24.1%), females (16.2%) and respondents of Indigenous identity (9.8%) were all more prone to experiencing familial breakdown.

*Mental illness*. With regards to mental illness, *age of respondent* (p ≤.05), *age at first homelessness* (p ≤.01), *Indigenous identity* (p ≤.05), and *health status* (p ≤.05) were all significantly related to ’mental illness’ as a barrier to housing (Table 5). Individuals under 25; both at the time of survey (31.0%) and when they first became homeless (20.8%), were more likely to report the inability to secure housing due to mental illness. Non-indigenous individuals (14.9%), those with poor health status (27.3%), and experience of foster care (21.8%) were also more likely than their counter-demographics to experience this barrier.

*Stigma/discrimination*. Only *age* (p ≤.01) and *age at first homelessness* (p ≤.05) were statistically related to *Barrier to housing*: *Stigma/discrimination*. The results in Table 5 indicate that those between the age of 35 and 44 (32.8%) were more likely than other age groups to experience stigma/discrimination-driven barriers to housing. The same was found for individuals who experienced homelessness for the first time before the age of 25 (28.7%).

### Multivariate analysis

#### Reasons for housing loss

The results of the multivariate analysis in Table 6 revealed that only *age of respondent* and gender were significantly associated to *Interpersonal/family issues* as a reason for housing loss. Individuals aged 25–34 were 72% less likely to experience *Interpersonal/family issues* compared to their counterparts aged under 25. *Gender* was significantly related to both *Housing/financial loss* and *Interpersonal/family issues* as the main reasons for most recent housing loss. Female individuals experienced *Housing/financial loss* and *Interpersonal/family issues* 2.2 times, and 2.5 times more than their male counterparts respectively did. *Indigenous status* and *health status* were not significantly correlated with any of the three reasons for housing loss. Respondents who received welfare income supports were 1.10, and 2.79 times more likely to experience housing/financial loss, and health-related housing loss than those who did not receive welfare income supports. Non-welfare respondents, however, were 39% more likely to experience interpersonal/family issues leading to homelessness. *Experience in foster care* was not significantly related to any of the three reasons for housing loss after adjusting for other characteristics. Finally, the selected characteristics in the regression analyses accounted for 11.1%, 13.5% and 16.8% of variance in *Housing/financial loss*, *Health related housing loss*, and *Interpersonal/family issues* as reasons for housing loss, respectively.

**Table 6:** Adjusted odds ratios (and 95% confidence intervals) from binary logistic regression of ever experiencing housing loss due to "housing/financial loss", "health issues", and "interpersonal/family issues" by selected characteristics among homeless individuals (N = 207), Nipissing District, Ontario 2021.

Characteristics	Housing/ financial loss	Health related	Interpersonal/ family issues
**Age**			
<25 (ref)	1.00	1.00	1.00
25–34	2.23 (0.62, 8.02)	1.56 (0.40, 6.11)	0.28 (0.08, 0.97)*
35–44	1.81 (0.49, 6.66)	0.47 (0.09, 2.27)	0.33 (0.09, 1.16)
45+	1.26 (0.33, 4.82)	1.11 (0.26, 4.73)	0.58 (0.16, 2.11)
**Gender**			
Male (ref.)	1.00	1.00	1.00
Female	2.19 (1.06, 4.54)*	0.96 (0.38, 2.46)	2.49 (1.19, 5.23)**
**Indigenous status**			
Non-Indigenous (ref)	1.00	1.00	1.00
Indigenous	1.34 (0.67, 2.67)	1.09 (0.48, 2.51)	0.65 (0.33, 1.28)
**Age at first homelessness**			
Less than 25 (ref)	1.00	1.00	1.00
25 and over	1.32 (0.60, 2.88)	0.62 (0.24, 1.58)	0.64 (0.30, 1.35)
**Health status**			
Poor (ref.)	1.00	1.00	1.00
Fair	2.27 (0.78, 6.55)	0.84 (0.26, 2.76)	1.22 (0.45, 3.33)
Good	1.10 (0.39, 3.05)	0.47 (0.15, 1.49)	2.36 (0.92, 6.07)
**Income source**			
Welfare	1.10 (0.40, 3.05)	2.79 (1.02, 7.64)*	0.61 (0.31, 1.21)
Non-welfare (ref)	1.00	1.00	1.00
**Experience in foster care**			
No (ref)	1.00	1.00	1.00
Yes	1.47 (0.62, 3.45)	1.11 (0.42, 2.97)	1.11 (0.42, 2.53)
**Nagelkerke R** ^**2**^	0.111	0.135	0.168

Ref. = Reference group; Significant level of Chi-square test:

*** p ≤ .001,

** p ≤ .01,

* p ≤ .05

#### Cumulative homelessness

The adjusted multivariate results in Table 7 show that those in older age groups are more likely to experience 180 or more days of homelessness each year. Specifically, those in the age groups of 25–34, 35–44, and 45+ are 7.9, 4.9, and 5.1 times more likely to experience chronicity than those under the age of 25. Those who became homeless at the age of 25 or above, were 49% less likely to experience chronic homelessness compared to their counterparts who became homeless under the age of 25. Respondents who receive welfare income supports were 3.28 times more likely to this experience. Other covariates were not significantly related with the odds of experiencing chronic homelessness, when controlling for other factors. These variables explained 23.0% of variance in cumulative homelessness within the Nipissing District of North-Eastern Ontario.

**Table 7:** Adjusted odds ratios (and 95% confidence intervals) from binary logistic regression of experiencing chronic homelessness (being homeless for 180 or more days in a given year) by selected characteristics among homeless individuals (N = 207), Nipissing District, Ontario 2021.

Characteristics	Odds Ratio (95% CI)
**Age**	
<25 (ref)	1.00
25–34	7.87 (2.06, 30.04)**
35–44	4.89 (1.23, 19.37)*
45+	5.09 (1.26, 20.57)*
**Gender**	
Male (ref.)	1.00
Female	0.62 (0.29, 1.33)
**Indigenous status**	
Non-Indigenous (ref)	1.00
Indigenous	1.15 (0.56, 2.35)
**Age at first homelessness**	
Less than 25 (ref)	1.00
25 and over	0.51 (0.23, 1.12)
**Health status**	
Poor (ref.)	1.00
Fair	0.96 (0.33, 2.78)
Good	0.76 (0.28, 2.05)
**Income source**	
Welfare	3.28 (1.56, 6.89)**
Non-welfare (ref)	1.00
**Experience in foster care**	
No (ref)	1.00
Yes	0.69 (0.29, 1.66)
**Nagelkerke R** ^**2**^	**0.230**

Ref. = Reference group; Significant level of Chi-square test:

*** p ≤ .001,

** p ≤ .01,

* p ≤ .05

#### Barriers to housing

Table 8 shows after adjusting for other characteristics, only *income source* was significantly (p ≤.05) related to *addiction* as a barrier to housing. Specifically, those who received welfare income supports were 2.92 times more likely to experience addiction as barrier to housing.

**Table 8:** Adjusted odds ratios (and 95% confidence intervals) from binary logistic regression of ever experiencing barriers to housing by selected characteristics among homeless individuals (N = 207), Nipissing District, Ontario 2021.

Characteristics	Addiction	Mental illness	Stigma/ discrimination
**Age**			
<25 (ref)	1.00	1.00	1.00
25–34	1.91 (0.49, 7.39)	0.32 (0.07, 1.38)	0.90 (0.26, 3.14)
35–44	1.45 (0.35, 6.06)	0.19 (0.04, 0.99)*	2.11 (0.59, 7.51)
45+	0.69 (0.15, 3.16)	0.14 (0.02, 0.78)*	0.39 (0.08, 1.74)
**Gender**			
Male (ref.)	1.00	1.00	1.00
Female	0.45 (0.17, 1.19)	0.48 (0.13, 1.74)	2.42 (1.02, 5.77)*
**Indigenous status**			
Non-Indigenous (ref)	1.00	1.00	1.00
Indigenous	1.06 (0.48, 2.37)	1.25 (0.43, 3.64)	1.16 (0.52, 2.56)
**Age at first homelessness**			
Less than 25 (ref)	1.00	1.00	1.00
25 and over	0.50 (0.21, 1.22)	0.78 (0.23, 2.61)	0.30 (0.12, 0.79)**
**Health status**			
Poor (ref.)	1.00	1.00	1.00
Fair	1.53 (0.46, 5.09)	0.09 (0.02, 0.43)**	0.96 (0.28, 3.27)
Good	0.72 (0.22, 2.34)	0.16 (0.05, 0.57)**	0.99 (0.32, 3.09)
**Income source**			
Welfare	2.92 (1.14,7.49)*	4.05 (1.06, 15.54)*	1.83 (0.77, 4.31)
Non-welfare (ref)	1.00	1.00	1.00
**Experience in foster care**			
No (ref)	1.00	1.00	1.00
Yes	0.67 (0.25, 1.80)	1.67 (0.52, 5.35)	1.06 (0.42, 2.65)
**Nagelkerke R** ^**2**^	**0.192**	**0.237**	**0.160**

Ref. = Reference group; Significant level of Chi-square test:

*** p ≤ .001,

** p ≤ .01,

* p ≤ .05

The results related to *Mental Illness* show that only age, health status, and income source were significantly related to experiencing *mental illness* as a barrier to housing (Table 8). Individuals aged 25 and older were less susceptible to housing barriers induced by mental illness. Compared with homeless individuals aged under 25, those aged 25–34, 34–44 and 45 and over were respectively 68%, 81% and 86% less likely to experience mental illness causing a direct barrier to housing. This pattern continued with first-time homelessness. Individuals who experienced homelessness after the age of 25 were 22% less likely than those before the age of 25 to face mental illness-related barriers to housing. Respondents who indicated they had fair or good overall health status were 91% and 84% less likely than those with poor overall health to suffer from mental illness-barriers to housing. Finally, the odds of reporting mental illness as a barrier to housing was 4.05 times higher for individuals who received welfare supports compared to those who did. The socioeconomic and demographic characteristics account for 23.7% of variation in the housing barriers induced by mental illness.

As the other housing barrier, stigma and/or discrimination-based barriers to housing were significantly related to *the respondent’s gender* and *age at first homelessness* (Table 8). Individuals who first became homeless at the age of 25 or over rather than before 25 were 70% less likely to experience stigma and/or discrimination that directly caused barriers to housing. Females were 2.4 times more likely than males to experience stigma and/or discrimination-based barriers to housing. The covariates in the model explained 16% of variance in the housing barrier of stigma and/or discrimination.

## Discussion

This study examined the socioeconomic and demographic factors associated with homelessness experiences during the 2021 COVID-19 pandemic in the Nipissing District in Ontario. The homelessness experiences examined were sleep location, chorionic and episodic homelessness, reasons for housing loss, and barriers to housing.

We found that younger respondents were more apt to stay in transitional shelters compared to their older counterparts. This result could be attributed to the existence of youth transitional housing located in North Bay. The ability for those under the age of 25 to stay in this transition house affords older individuals the opportunity to access emergency shelters in the area amid reduced bed availabilities, further explaining the increased use of emergency shelters among older adults. It was also found that those under the age of 35 were more commonly found in institutional settings, such as correctional facilities. The same was found with male respondents. These findings could be due to higher crime rates among these demographic sub-groups [41]. Individuals with experience in foster care were more likely to access homeless shelter supports, including emergency shelters, transitional housing, and institutional settings. This increased likelihood could perhaps be a direct result of unpreparedness for transitions out of institutionalized care, and lack of life-skills development [19, 42]. Finally, a most interesting finding with regards to sleeping location was the increased number of respondents who indicated they would be sleeping in an unsheltered public location. This result could be due to reduced shelter capacities introduced during the COVID-19 pandemic.

The bivariate and multivariate results showed that younger individuals were significantly less likely to experience chronic homelessness compared to their older counterparts. With knowledge from a previous trend analysis [2] that first-time homelessness is becoming increasingly more frequent among those over the age of 45, these results suggest that not only are older individuals experiencing homelessness for the first time, but they are staying homeless for prolonged periods of time. The results from the analysis of episodic homelessness further support this with the finding that older respondents are experiencing single episodes of homelessness compared to their younger counterparts. This could be due to a rise in unemployment rates that was triggered by the pandemic, as businesses began operating with reduced staff to maintain social distancing protocols [2]. Similar results are found in the United States, where the pandemic has been cited to be a large contributor to the recession started in 2019 resulting in “steep job losses” [43]. The results also revealed that male individuals, and those receiving welfare income supports are more susceptible to experiencing chronic homelessness. This is congruent with previous studies [1–5, 7, 9, 27, 37, 40, 42, 44], noting that the COVID-19 pandemic is likely not at play. Welfare recipients were also more likely to experience health-related housing loss, addiction, and mental health-based barriers to housing. These findings could be attributed to the structural factors explored above. This includes systematic problems associated with poor public planning and policies that increase the number of people living in marginal economic and housing circumstances, and the supply and demand of safe and affordable housing [7, 10–12]. The lack of housing options accessible to these marginalized groups has been highlighted throughout this research as a prominent barrier, and further supports the need for advocacy for increased welfare income support, and the development of safe and affordable housing options. This need has significantly increased since the beginning of the pandemic, with a spike in living costs [40, 45], unaccompanied by an increase in social welfare amounts. A single individual receiving Ontario Disability Support Program (ODSP) is eligible for $672.00 for basic needs, and $497.00 in shelter allowances for a total of $1,169.00 monthly [17]. This amount is even less for recipients of Ontario Works (OW) where singles are eligible for $343.00 for basic needs, and $390.00 in shelter allowances for a total of $733.00 monthly [17]. These income supports are not sufficient when compared to the inflation of housing costs seen over the last year. It has been noted that average rent prices in Canada have increased by 3% in 2021 [34]. Currently, a one-bedroom apartment in North Bay, Ontario rents for over $1,300.00 monthly [35]. Similar trends can be observed in the United Kingdom, where private housing rental prices have increased by 4% since the beginning of the pandemic [46]. These prices make the idea, and reality of affordable housing unsustainable to welfare income recipients. These combined results support the final stage in the “Cycle of Homelessness” [7] noting that the homeless experience is near-impossible to escape. It is now clear that this stage of the cycle not only occurs on the individual level, but to the extent of the service system working to eliminate homelessness itself.

Results showed that female individuals were roughly twice as likely to experience housing/financial loss, or interpersonal/family issues directly causing homelessness compared to their male counterparts. Further, females were at an increased risk of experiencing family breakdown-related barriers to housing. These results are consistent with previous studies conducted across Ontario [4, 7], and could be attributed to the increased (3.5 times) likelihood of females to experience domestic victimization from their partner/spouse [18, 38], fleeing as a result. This finding supports the reviewed literature, claiming that the result of fleeing domestic violence or the abrupt end of a relationship can act as a precursor to homelessness [18, 37]. These findings could be attributed to increased durations of time spent in “lockdown” with family members and/or partners [38]. Respondents under the age of 25 were found to experience interpersonal/family conflict that directly caused their most-recent housing loss at an increased rate as well, along with family breakdown, addiction, and mental illness-rooted barriers. This is similar to findings from the United States where ‘family breakdown’ is cited as a predominant cause of youth homelessness [47]. These results suggest that those under 25 have become homeless due to parental conflict and developed mental illness or substance use that has evolved into addiction as a result. This sequence of events supports the conceptual model well, where young people (who are potentially homeless) experience an adverse life event that ultimately propels them into homelessness (parental conflict), and consistently experience barriers that prevent them from finding adequate housing solutions [8].

## Strengths and limitations

As an innovative approach to homelessness research in the District of Nipissing, this study applied the “Cycle of Homelessness” conceptual framework [7] to a sub-population of homelessness that has previously not been examined in such detail. As a result, this research was able to provide insight and context to the unique population of individuals experiencing homelessness in the Northeast region of Ontario, Canada, including demographic composition and current parameters that work together to create susceptibility to this social phenomenon. This research can influence and support the development or dissolution of social policies and programs related to housing and homelessness in the Nipissing District. Although the findings of the study could be considered the most current reliable analyses of homelessness in the District of Nipissing, Ontario, limitations may be addressed in future studies. The data gathered from close-ended surveys are not so rich and in-depth that one can interpret the rationales behind the complex process of homelessness. Therefore, research with a qualitative approach could perhaps have accomplished this goal.

## Conclusion

This study examines sociodemographic risk factors and experiences that drive the “Cycle of Homelessness” in the District of Nipissing, Ontario, Canada. Our analyses reveal an increase in the number of people sleeping in unsheltered locations, due to reduced shelter capacities and the need for social distancing. We also discovered that younger respondents were more often found using transitional housing options compared to their older counterparts, because of the existing youth transitional shelter in North Bay. This freed up emergency shelter beds for those over the age of 25 –a significant impacting factor during the pandemic. Our results also identified that individuals are becoming homeless for the first time at an older age and remaining homeless for prolonged periods of time compared to their younger counterparts. However, these younger individuals are experiencing increased numbers of episodic homelessness. Gender differences in homeless experiences also showed males to be more susceptible to chronic homelessness, and females are more apt to face housing loss related to financial hardship or interpersonal conflict. Our findings show that the old model of the “Cycle of Homelessness” is still applicable to explaining correlates of homeless experiences in the Nipissing District. Perhaps the most pressing finding of this study showed welfare recipients to experience health-related housing loss, chronic homelessness, addiction, and mental health-based barriers to housing. These findings could be attributed to structural factors associated with poor public planning and policies that increase the number of individuals living in marginal economic and housing circumstances, especially during the COVID-19 pandemic. This study therefore advocates for increased welfare income supports and identifying lack of sustainable housing options accessible to marginalized groups as a main barrier to eliminating homelessness.

## Supporting information

S1 Checklist Human participants research checklist. (DOCX)
